# Joint Access Authentication and Task Collaboration for Dynamic Cross-Domain Resource Management in Satellite Networks

**DOI:** 10.3390/s26175450

**Published:** 2026-08-28

**Authors:** Jiali Zhu, Ning Zhang, Yang Yang, Yuan Yao, Weiwei Qi, Yun Wang

**Affiliations:** 1 Nanjing Marine Radar Research Institute, Nanjing 210000, China; 2 School of Computer Science and Engineering, Southeast University, Nanjing 211189, China; ywang_cse@seu.edu.cn; 3 Key Laboratory of Computer Network and Information Integration, Ministry of Education, Southeast University, Nanjing 211189, China

**Keywords:** satellite network, authentication, resource management, domain

## Abstract

Low Earth Orbit (LEO) satellite systems provide wide-area Earth coverage and constitute an important component of the integrated space–air–ground network. With the increasing demand for near-data real-time task processing at the near-data end, constructing a reliable resource management solution is crucial to guaranteeing efficient task execution. Considering the frequent networking due to high-speed movement, unbalanced dynamic task allocation, and unknown resource requirements, we propose a hierarchical dynamic cross-domain collaborative resource management architecture considering satellite access. To enable low-cost access authentication over narrowband inter-satellite links, this work proposes a non-interactive privacy-preserving authentication scheme. Each satellite aggregates four committed orbital attributes into one compact proof, which can be verified by multiple authorized satellites within a domain and epoch. Concurrent access requests are handled via verifier-side batch verification of independent proofs. Additionally, considering the imbalance and dynamics of satellite task allocation, a hierarchical resource management scheme combining multi-agent reinforcement learning intra-domain resource allocation and inter-domain early-exit matching game task collaboration is proposed to alleviate the differences in inter-domain task allocation and enable efficient task execution under limited satellite resources. Simulation results show that the proposed scheme has lower overall communication overhead and an improved task completion rate in dynamic scenarios.

## 1. Introduction

Low Earth Orbit (LEO) satellite communication systems are becoming a key communication network paradigm for various applications in remote area communications, navigation, weather monitoring, and military missions [1,2,3,4,5]. From the point of view of the difference in satellite orbital altitude, LEO satellites, compared with geostationary satellites, which are at a constant orbital altitude of 35,786 km, have the advantages of high maneuverability, low energy consumption for communications, low latency, and flexible deployment. They are the core medium for maintaining data interaction and communication links between the ground users and the network control center [6]. However, due to the inherent characteristic of low orbital altitude, LEO satellite communication systems require hundreds or even thousands of satellites to meet the demand for global communication coverage in the form of satellite organization in the same-altitude orbital plane or a hierarchical communication architecture. Many government agencies and commercial companies have launched large-scale satellite constellation programs with different functional designs, such as Starlink, Kuiper, GW Constellation, and OneWeb [7,8,9].

As a key communication component of non-terrestrial networks, LEO satellite communication systems need to achieve self-organized and efficient networking and onboard mission execution without relying on the assistance of terrestrial infrastructure. Satellite authentication is a prerequisite for secure network service access and inter-satellite mission collaboration [10]. Considering the open and hostile environment of satellite deployment, which poses the risk of eavesdropping on fragile inter-satellite links [11], the difficulty of maintaining long-time stable connectivity on frequently switched dynamic links [12], and the limited supply of equipment capacity that restricts the complexity of the authentication protocol implementation [13], there is an urgent need for a fast satellite authentication scheme that meets the requirements of sensitive data protection, low overhead, and concurrent processing of independent authentication requests from multiple satellites. In addition, due to the spatial and temporal imbalance of satellite mission distribution, the unpredictability of instantaneous resource availability, and the need for efficient scheduling of tasks with different priorities, it is urgent to find a practical satellite-mission bilateral resource management framework.

In this paper, we focus on the domain communication system of LEO satellites at the same orbital altitude using a virtual topology algorithm. Satellite motion is a continuous dynamic process. Time discretization modeling is employed to lower simulation complexity. Topology stays stable inside each time slot, and the virtual topology is updated across time slots based on orbital motion. Different LEO satellites construct domains and establish communication networks based on geographic locations. Compared with conventional key-management-based authentication schemes [14], the proposed zero-knowledge-proof (ZKP)-based [15] authentication scheme achieves verification for any third party within the network without exposing sensitive information, without requiring strong trust assumptions such as trusted hardware or shared keys. Reinforcement learning [16] provides an effective alternative to conventional static resource-management strategies for solving the resource allocation problem in dynamic environments by using accumulated, learned action experience and environmental information to optimize behavioral selection strategies. Specifically, the main contributions of this paper are as follows:

1. A non-interactive privacy-preserving satellite access authentication scheme based on orbital information is proposed. For each authenticated satellite requesting network access, four committed orbital attributes, including the semi-major axis, orbital inclination, mean anomaly, and right ascension of the ascending node, are jointly proven through a single aggregated Bulletproof. The resulting proof is generated once and can be independently verified by multiple authorized verifier satellites within the same domain and authentication epoch. When several satellites request access concurrently, they generate independent proofs, which are processed through verifier-side batch or parallel verification. The proposed scheme does not assume multi-satellite joint proof generation.
2. A dynamic hierarchical domain collaborative resource management framework considering limited buffers, unpredictable tasks, etc., is proposed, which combines intra-domain resource management strategy and inter-domain task collaboration strategy; the intra-domain multi-satellite resource management strategy uses a reinforcement learning scheme based on actor-critic networks with offline centralized training and online decentralized execution to maximize the local task completion rate. The inter-domain task collaboration strategy uses an early-exit matching game strategy to construct a stable two-way selection collaboration between domains based on matching preference rules to achieve flexible allocation of available resources and optimization of resource utilization.
3. A simulated low-orbit satellite communication system is constructed and extensively simulated and analyzed. The computational and communication overheads of the proposed scheme for dynamic satellite access authentication are explored, and parameters such as the number of tasks and the rate of task generation are varied to study the reliability of the hierarchical domain cooperative resource management scheme in different scenarios. In addition, an in-depth comparative performance analysis of related studies is carried out.

Successful authentication is not treated as an isolated security procedure. Instead, it directly determines whether a satellite can join the virtual resource pool. Only authenticated satellites contribute computation, storage, and transmission resources, and the updated resource state subsequently drives the reinforcement-learning scheduler and the inter-domain matching process.

The remainder of the paper is structured as follows. Section 2 reviews and analyses related research. Section 3 elaborates on implementing a dynamic, intelligent resource management scheme for joint zero-knowledge authentication and domain task collaboration. Section 4 provides a theoretical analysis of the robustness and security of the proposed protocol. A comparative analysis of the implementation performance and overhead cost of the proposed scheme, as well as previous studies, is presented in Section 5. Section 6 provides the conclusion.

## 2. Related Work

This section describes the authentication scheme for satellite communication systems and then the resource management scheme.

### 2.1. Authentication Scheme

Various authentication schemes have been proposed for LEO satellite communication systems to address the challenges encountered in practical deployment. Centralized authentication and key management schemes rely on terrestrial infrastructure entities to provide authentication credential management, and Lv et al. [17] propose an LEO satellite IoT-based authentication and key management framework for the Internet of Things. However, the requirement for continuous session-key exchange inevitably increases energy consumption, resulting in a shorter device lifetime. Lightweight authentication schemes aim to reduce communication and computational overhead while maintaining security; Khan et al. [18] propose a two-phase authentication strategy using pseudo-random binary access time slots, which, unfortunately, is prone to user data discrepancies due to synchronization delays. To alleviate the inherent shortcomings of centralized authentication schemes for satellite networks, Khan et al. [19] proposed a decentralized continuous authentication framework using a decentralized key management process to alleviate the burden of synchronization equipment due to frequent switching of satellites in orbit.

Unlike previous research on cryptographic satellite network access authentication based on public key infrastructures (PKIs), a class of low-complexity authentication mechanisms called physical layer authentication that exploits the physical characteristics of the wireless channel or the characteristics of the hardware device to provide the device with a non-cloneable identity has been proposed. As an authentication scheme based on channel characteristics, Topal et al. [20] proposed a scheme that uses the continuous Doppler shift due to the high mobility of satellites to differentiate legitimate satellite identities, which does not need to feed back the channel information but needs to rely on the assumption that LEO satellites are assumed to share mobility information. In addition, device feature authentication schemes that treat hardware impairments as unique identifiers [21,22] are also partially studied.

In other communication networks (vehicular self-organizing networks), an authentication protocol based on the ZKP approach called an adaptive group-based zero-knowledge proof authentication protocol [23] has been proposed. This scheme also does not rely on computationally expensive password-based authentication. It prevents network entities from using authentication information to track user activities by adding dynamic update parameters to the private authentication information. In addition, several works [24,25] have attempted to improve the reliability of the authentication process by combining consensus protocols and on-net authentication, including the common Raft and Practical Byzantine Fault Tolerance (PBFT) consensus protocols.

### 2.2. Resource Management Scheme

Effective resource management helps to improve the mission execution capability of satellite communication systems. In general, collaborative task offloading is modeled as a multidimensional joint resource management problem, and these schemes [26,27,28] individually or partially consider factors such as time-varying network topology, task execution resource requirements, and task execution time constraints and develop improved iterative optimization algorithms to obtain the optimal solution under some of the condition constraints. However, increasing constraints increases the overhead of solving the optimization problem. Conflicts among these constraints are difficult to resolve simultaneously. More importantly, this type of scheme is not well adapted to the task offloading problem in dynamic environments, especially considering the unpredictable task scenarios.

To incentivize entities to collaborate fairly, Gao et al. [29] use a Nash bargaining game in a multi-user multidimensional resource management problem to assign matching throughputs to users with different quality of service requirements. Similarly, Mi et al. [30] constructed an intelligent matching resource management framework for the dynamic management of network resources, considering the differences in the demand for different kinds of resources in specific task execution. They used the Gale and Shapley matching game to reduce the computational complexity during the matching game. However, the game-based resource management scheme requires frequent interactions between entities until both matching parties are challenged to be preempted by the more preferred entities, and the process is accompanied by serious interaction communication overheads that increase exponentially with the number of entities involved in matching.

Recently, deep reinforcement learning has been widely adopted for satellite resource management; a class of resource management schemes using deep learning has been proposed, one after another. To study the resource allocation problem of multi-beam star-ground networks, He et al. [31] proposed a multi-agent reinforcement learning scheme that compromises communication delay and bandwidth consumption. This centralized training and decentralized execution reinforcement learning scheme can be used to adapt to the resource management problem in dynamic environments efficiently, but in order to enhance the algorithm’s generalization ability in unknown scenarios, Geng et al. [32] used meta-learning for unsupervised learning of graph neural networks with different traffic distributions. Many existing schemes ignore the resource management problem of cross-domain collaboration while considering random task traffic and unbalanced task distribution.

Although Hu et al. [15] also employ zero-knowledge techniques for satellite access authentication, several fundamental differences exist. First, Hu et al. [15] focus on proving a single authentication statement, whereas our scheme aggregates multiple orbital-attribute range statements of one satellite into a single bulletproof. Second, our protocol explicitly binds proofs to registered orbital commitments and authentication context (domain, epoch, and session identifier), preventing cross-identity proof reuse. Third, authenticated satellites are immediately incorporated into the virtual resource pool, establishing a direct coupling between authentication outcomes and subsequent resource scheduling.

## 3. Dynamic Cross-Domain Resource Management Scheme

**Figure 1:**
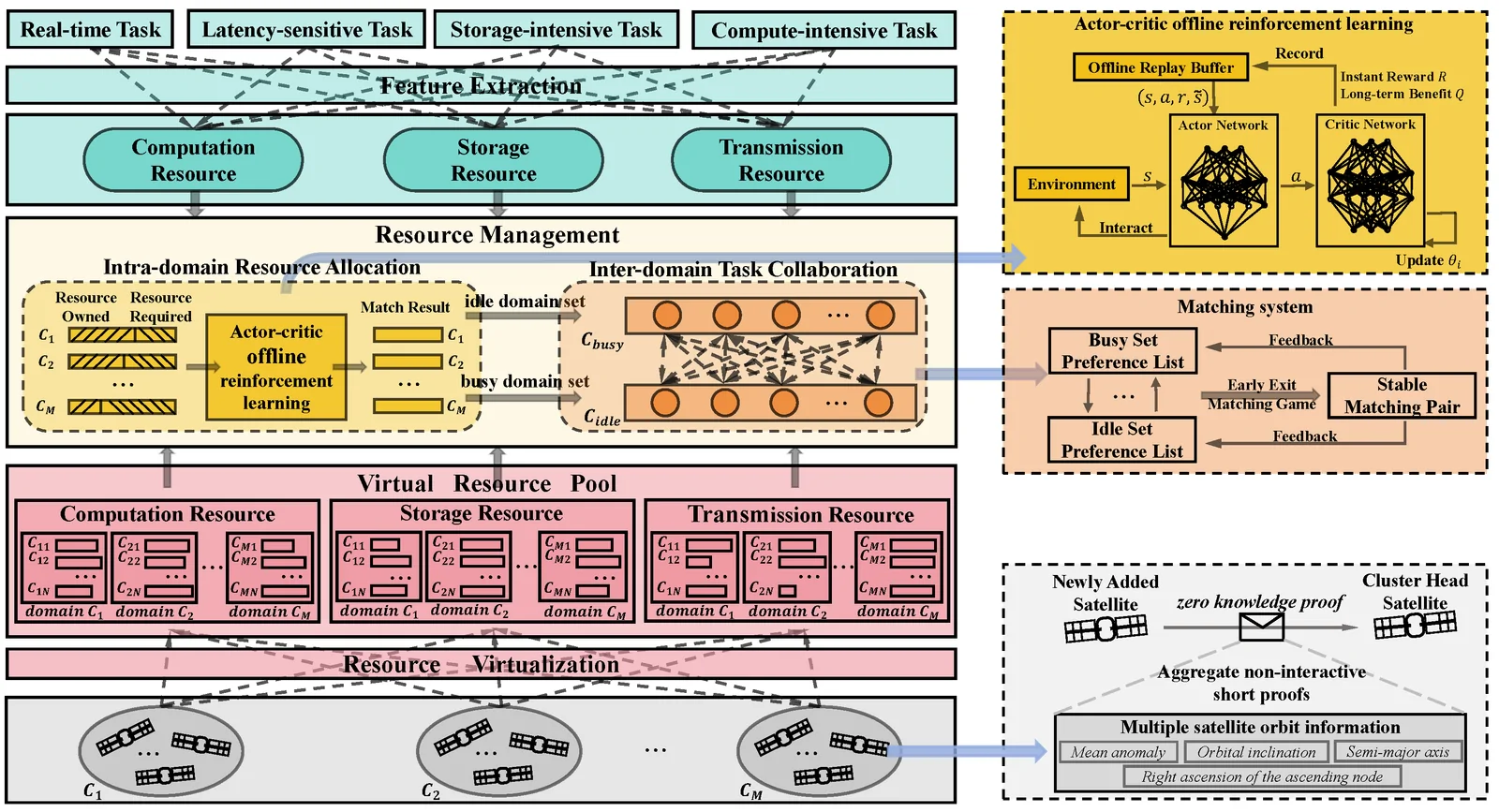
Cross-domain dynamic resource management policy architecture.

This section presents the proposed cross-domain dynamic resource-management framework, as illustrated in Figure 1. The architecture consists of an infrastructure layer (grey), a resource virtualization layer (pink), a resource management layer (orange), and an application layer (green), respectively, from top to bottom. The LEO satellites in the infrastructure layer are divided into domains based on the geographical location of their orbits, and non-interactive zero-knowledge identity verification is performed for the satellites requesting access to the domain. Simultaneously, a cross-domain collaborative satellite resource allocation scheme is implemented in response to virtualized network mission resource requirements. The proposed protocol assumes that the registered Pedersen commitments are generated during a trusted satellite enrollment phase and are maintained by the domain management system. Therefore, the zero-knowledge proof verifies possession of a valid opening for a previously registered commitment rather than establishing authorization from arbitrary orbital parameters.

### 3.1. Non-Interactive Zero-Knowledge Satellite Authentication

In the LEO satellite communication system, facing the new entry of LEO satellites into the network, verifying the identity legitimacy of the necessary access devices before establishing communication and synchronizing data is necessary. In order to meet the communication requirements of narrow bandwidth and weak-link wireless networks, we propose to use a lightweight privacy-preserving authentication scheme supporting single-satellite multi-attribute aggregation and verifier-side batch processing of independent proofs. Bulletproofs [33] have attracted attention for their non-disclosure of committed information, lack of trusted setups, and logarithmic proof scale.

**Figure 2:**
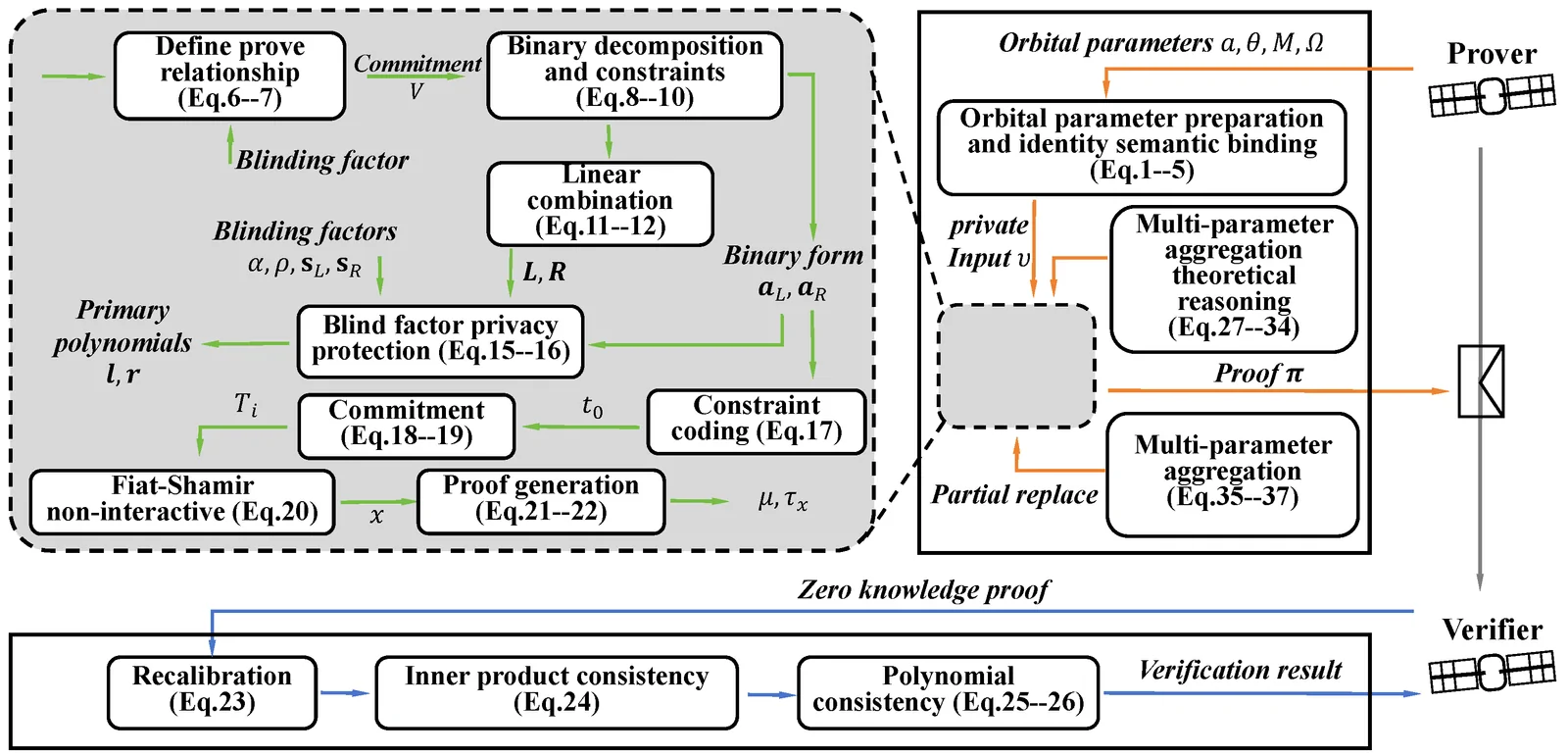
Authentication protocol flowchart.

This section reconstructs a single-submission, independently verifiable non-interactive zero-knowledge authentication proof. The protocol first reduces the legitimacy of orbital parameters to interval constraints and bit consistency constraints, verifying that the orbital parameters held by the Prover fall within reasonable physical ranges rather than relying on direct trusted identities. To prevent the reconstruction of orbital parameters from eavesdropped information, a commitment mechanism incorporating blinding factors is employed to privatize intermediate sensitive information that would otherwise be directly exposed for verification purposes. Without requiring the online generation of random challenges, the Fiat–Shamir heuristic is utilized to transform challenge generation into a deterministic derivation from commitments via a hash function, enabling offline verification with a single submission. Further adapting to narrowband constraints, the scheme aggregates the range relations of multiple orbital attributes belonging to one satellite into a single proof. The size of the compressed inner-product proof grows logarithmically with the aggregated vector dimension, whereas proof generation and verification still require linear-size multi-scalar computations. Specifically, the workflow of each module in the complete authentication protocol and their corresponding relationships with formula derivations are referred to in Figure 2.

A satellite declares its legitimacy by generating a zero-knowledge proof over orbit-information-bound authentication data derived from its orbital parameters, rather than using the orbital parameters themselves as identity credentials. The security of the proposed authentication protocol does not rely on the confidentiality of the orbital parameters themselves, but on the binding property of the Pedersen commitments, the randomness introduced by independent blinding factors, and the soundness of the Bulletproof proof system. Therefore, even if an adversary can estimate or observe plausible orbital parameters, it cannot construct a valid authentication proof without the corresponding commitment randomness and a valid witness satisfying the proof relations. Therefore, the proposed authentication scheme is executed whenever a satellite joins a new virtual topology or requires cross-domain access authentication. To describe the protocol flow, simply ignoring orbital eccentricity, an ideal circular orbit requires only four elements to uniquely determine a satellite’s position in orbital space: semi-major axis *a*, orbital inclination angle θ, mean anomaly *M*, and right ascension of the ascending node Ω. The orbital parameters are used for orbit-information binding rather than precise orbit propagation. Nevertheless, for satellite systems operating on highly elliptical orbits or applications requiring high-precision orbital descriptions, the eccentricity parameter can be incorporated into the orbit-information vector without affecting the overall protocol architecture or the security analysis. In the Perifocal Coordinate System (PQW), the mean anomaly *M* approximates a circular satellite orbit, and the spatial coordinates of the satellite are expressed as follows:

(1) $$x_{PQW}=r\cos M=a\cos M,$$

(2) $$y_{PQW}=r\sin M=a\sin M,$$

(3) $$z_{PQW}=0.$$

The corresponding position in the Earth-Centered Inertial (ECI) frame is obtained through the following simplified rotation matrix:

(4) $$R=\left[\begin{array}{ccc} \cos\Omega & -\sin\Omega & 0\\ \sin\Omega & \cos\Omega & 0\\ 0 & 0 & 1 \end{array}\right]\left[\begin{array}{ccc} 1 & 0 & 0\\ 0 & \cos\theta & -\sin\theta \\ 0 & \sin\theta & \cos\theta \end{array}\right].$$

The position of the satellite in the ECI coordinate system is expressed as:

(5) $$\left[\begin{array}{c} x_{ECI}\\ y_{ECI}\\ z_{ECI} \end{array}\right]=R\left[\begin{array}{c} x_{PQW}\\ y_{PQW}\\ z_{PQW} \end{array}\right]=\left[\begin{array}{c} a\left(\cos\Omega \cos M-\sin\Omega \cos\theta \sin M\right)\;\\ a\left(\sin\Omega \cos M+\cos\Omega \cos\theta \sin M\right)\;\\ a\sin\theta \sin M\; \end{array}\right].$$

Let G be a cyclic group of prime order *p*, and let $Z_{p}$ denote the finite field of integers modulo *p*. The public parameters include independent generators *g*, $h_{0}$, u∈G and generator vectors $g=\left(g_{1},\cdots ,g_{N}\right)$, $h=\left(h_{1},\cdots ,h_{N}\right)\in G^{N}$. Here, *g* and $h_{0}$ are used for scalar Pedersen commitments, g and h are used for vector commitments, and *u* is used in the inner-product argument. All exponents are elements of $Z_{p}$. Bold lowercase symbols denote vectors. For $a,b\in Z_{p}^{N}$, their inner product and Hadamard product are defined as $\langle a,b\rangle =\sum_{i=1}^{N}a_{i}\cdot b_{i}$, $a\circ b=\left(a_{1}b_{1},\ldots ,a_{N}b_{N}\right)$. Vector exponentiation is written as $g^{a}=\prod_{i=1}^{N}{g_{i}}^{a_{i}}$. We further define $1^{N}=\left(1,\ldots ,1\right)$, $2^{n}=\left(1,2,\ldots ,2^{n-1}\right)$, $y^{N}=\left(1,y,\ldots ,y^{N-1}\right)$. The physical orbit attributes are first quantized into non-negative integers before being used as witnesses. The symbol υ denotes one quantized attribute value, whereas *a* continues to denote the physical semi-major axis.

Taking the semi-major axis a of the orbiting satellite as an example, as the private input υ of the satellite to be netted (Prover), the constructed commitment proves the following range relation, namely:

(6) $$R_{rng}^{\left(n\right)}=\left\{\left(V;\upsilon ,\gamma \right)\right.\left.|V=g^{\upsilon }h_{0}^{\gamma }\wedge \upsilon \in \left[0,2^{n}\right]\right\},$$

where γ is a blinding factor to improve the privacy of the commitment *V*. The prover demonstrates that it knows an opening of the fixed public commitment *V* to an *n*-bit non-negative integer. The corresponding range relation is defined as follows:

(7) $$V=g^{\upsilon }h_{0}^{\gamma },\;\upsilon ,\gamma \in Z_{p},$$

(8) $$\langle a_{L},2^{n}\rangle =\upsilon ,\;a_{L}\in {\left\{0,1\right\}}^{n},$$

(9) $$a_{R}=a_{L}-1^{n},$$

(10) $$a_{L}\circ a_{R}=0^{n}.$$

Instead of checking the bit-decomposition, reconstruction, and bitness constraints separately, the prover and verifier combine them through the scalar challenges $y,z\in Z_{p}^{*}$. The scalar *y* determines the public power vector $y^{n}=\left(1,y,\ldots ,y^{n-1}\right)$. The combined constraint is written as follows:

(11) $$\left(\langle a_{L},2^{n}\rangle -\upsilon \right)\cdot z^{2}+\langle a_{L}-a_{R}-1^{n},y^{n}\rangle \cdot z+\langle a_{L},a_{R}\circ y^{n}\rangle =0,$$

Define $l_{0}=a_{L}-z1^{n}$ and $r_{0}=y^{n}\circ \left(a_{R}+z1^{n}\right)+z^{2}2^{n}$. The combined constraint can then be represented by the following inner-product identity:

(12) $$\langle l_{0},r_{0}\rangle =z^{2}\upsilon +\delta_{n}\left(y,z\right).$$

It is worth noting that $\delta_{n}\left(y,z\right)=\langle 1^{n},y^{n}\rangle \left(z-z^{2}\right)-z^{3}\langle 1^{n},2^{n}\rangle$. $l_{0}$ and $r_{0}$ still depend directly on the witness. Revealing these vectors would expose witness-related information and would also result in a proof whose size grows linearly with *n*. To hide the witness-dependent vectors, the prover independently samples $\alpha ,\rho \overset{a}{\leftarrow }Z_{P}$, $s_{L},s_{R}\overset{⁠}{\leftarrow }Z_{P}^{n}$, and constructs the vector Pedersen commitments *A* and *S* as follows:

(13) $$A=h_{0}^{\alpha }g^{a_{L}}h^{a_{R}},$$

(14) $$S=h_{0}^{\rho }g^{s_{L}}h^{s_{R}}.$$

Commitment *A* binds the binary-decomposition vectors while hiding them through α. Commitment *S* commits to independent random mask vectors and is blinded by ρ. After the challenges *y* and *z* are determined, the prover forms the following vector polynomials:

(15) $$l\left(X\right)=a_{L}-1^{n}z+s_{L}X,$$

(16) $$r\left(X\right)=y^{n}\circ \left(a_{R}+z1^{n}+s_{R}X\right)+z^{2}2^{n}.$$

In the interactive zero-knowledge proof scheme, the Prover must first declare its generated commitments *A*, *S* to the Verifier and obtain the random factors *z*, y generated by the Verifier. To avoid additional communication rounds, the Fiat–Shamir transformation deterministically derives the challenges from the public transcript and the session context. Let $ctx=\left\{domainID,epoch,sid\right\}$, where domainID identifies the target domain, epoch specifies the valid time window and sid is a session identifier. The context contains no verifier-specific identifier so that multiple authorized verifiers in the same domain can independently verify the same transcript. The challenges are derived sequentially as $z=RO\left(A,S,ctx\right)$ and $y=RO\left(A,S,z,ctx\right)$.

The coefficients of $l\left(X\right)$ and $r\left(X\right)$ are vectors in $Z_{p}^{n}$. The construction of the polynomial *t* needs to satisfy that $\upsilon \in \left[0,2^{n}\right]$ and $t_{0}$ maintains a particular form, i.e.,

(17) $$\begin{array}{c} t_{0}=\langle a_{L},a_{R}\circ y^{n}\rangle +z\langle a_{L}-a_{R},y^{n}\rangle +z^{2}\langle 2^{n},a_{L}\rangle +\delta_{n}\left(y,z\right)\\ \;=z\langle 1^{n},y^{n}\rangle +z^{2}\langle a_{L},2^{n}\rangle +\delta_{n}\left(y,z\right)=z\langle 1^{n},y^{n}\rangle +z^{2}\upsilon +\delta_{n}\left(y,z\right), \end{array}$$

where $k\left(y,z\right)=-z^{2}\langle 1^{n},y^{n}\rangle -z^{3}\langle 1^{n},2^{n}\rangle$. If $t_{0}$ satisfies the above formulation, then it can be verified that the relationship $a_{L}$ and $a_{R}$ holds. Prover constructs the second-order primary polynomials l, r and computes the

(18) $$\hat{t}=t\left(X\right)=\langle l(X),r(X)\rangle =t_{0}+t_{1}X+t_{2}X^{2}.$$

Since both l(X) and r(X) are linear in *X*, their inner product is a polynomial of degree two. The prover samples $\tau_{1},\tau_{2}\in Z_{p}$ and commits to the non-constant coefficients $t_{1}$ and $t_{2}$:

(19) $$T_{i}=g^{t_{i}}h_{0}^{\tau_{i}},i\in \left\{1,2\right\},$$

(20) $$x=RO\left(T_{1},T_{2},ctx,A,S,y,z\right),$$

(21) μ=α+ρx,

(22) $$\tau_{x}=\tau_{1}x+\tau_{2}x^{2}+z^{2}\gamma ,$$

Function $RO\left(\cdot \right)$ denotes a cryptographic hash function implementing the Fiat–Shamir transform to convert the interactive proof into a non-interactive one. $RO\left(\cdot \right)$ is instantiated using SHA-256, whose 256-bit output is used to derive the challenge value from the public proof transcript. The Verifier can perform the following process to verify that the proof is correct.

(23) $$h_{i}^{\prime }=h_{i}^{y^{-i+1}},i=1,\ldots ,n,$$

(24) $$\hat{t}\overset{?}{=}\;\langle l,r\rangle ,$$

(25) $$g^{\hat{t}}h_{0}^{\tau_{x}}\overset{?}{=}\;g^{\delta_{n}\left(y,z\right)}V^{z^{2}}T_{1}^{x}T_{2}^{x^{2}},$$

(26) $$P=AS^{x}\cdot g^{-z1^{n}}{\left(h^{\prime }\right)}^{zy^{n}+z^{2}2^{n}}={h_{0}}^{\mu }g^{l}{\left(h^{\prime }\right)}^{r}.$$

Let $P_{\mathrm{IP}}=Pu^{\hat{t}}=g^{l}{\left(h^{\prime }\right)}^{r}u^{\langle l,r\rangle }$. Acceptance implies that the prover knows vector openings consistent with the fixed commitment and the prescribed range relation; it does not mean that the verifier explicitly calculates the hidden value υ. The basic relation in Equation (6) proves only that a committed integer belongs to $\left[0,2^{n}\right)$. In the satellite scenario, the *j*-th quantized attribute is required to satisfy a physical interval $L_{j}\leq \upsilon_{j}\leq U_{j}$. To prove both bounds without introducing unrelated commitments, the lower- and upper-bound offset commitments are deterministically derived from the registered commitment. The lower-bound offset is defined as follows:

(27) $$W_{j,-}=V_{j}g^{-L_{j}}=g^{\upsilon_{j}-L_{j}}h_{0}^{\gamma_{j}},w_{j,-}=\upsilon_{j}-L_{j},$$

Because $W_{j,-}=g^{\upsilon_{j}-L_{j}}h_{0}^{\gamma_{j}}$, proving that its committed value belongs to $\left[0,2^{n_{j}}\right)$ establishes the lower-bound condition, provided that $2^{n_{j}}>U_{j}-L_{j}$.

(28) $$W_{j,+}=g^{U_{j}}V_{j}^{-1}=g^{U_{j}-\upsilon_{j}}h_{0}^{-\gamma_{j}},w_{j,+}=U_{j}-\upsilon_{j}.$$

When both bounds are enforced for each of the four orbit-related attributes, the aggregated proof contains q=2m=8 range values rather than m=4.

The vector relation in Equation (26) still has dimension *n*, and directly revealing its openings would produce a linear-size transcript [34]. To avoid confusion with the scalar value commitment *V*, the commitment used by the inner-product argument is denoted by $P_{\mathrm{IP}}$. The corresponding relation is defined as follows:

(29) $$P_{\mathrm{IP}}=\left\{\left(P_{\mathrm{IP}},g,h^{\prime },u;l,r\right)|P_{\mathrm{IP}}=g^{l}{\left(h^{\prime }\right)}^{r}u^{\langle l,r\rangle }\right\}.$$

Here, l and r are the private vector openings, whereas $P_{\mathrm{IP}}$, g, $h^{\prime }$ and *u* are public inputs to the inner-product verification. At each recursion round, the current vectors and generator vectors are divided into left and right halves. If the initial aggregated dimension is not a power of two, the vectors are padded with zeros to the next power-of-two dimension before recursion.

(30) $$Map\left(a_{L},a_{R},b_{L},b_{R},c\right)=g_{L}^{a_{L}}g_{R}^{a_{R}}h_{L}^{b_{L}}h_{R}^{b_{R}}u^{c},$$

(31) $$d^{\prime }=\frac{d}{2},$$

(32) $$P_{\mathrm{IP}}=g_{L}^{l_{L}}g_{R}^{l_{R}}{h^{\prime }}_{L}^{r_{L}}{h^{\prime }}_{R}^{r_{R}}u^{\langle l_{L},r_{L}\rangle +\langle l_{R},r_{R}\rangle }.$$

The prover computes the cross-inner-product commitments

(33) $$L=g_{R}^{l_{L}}{\left(h_{L}^{\prime }\right)}^{r_{R}}u^{\langle l_{L},r_{R}\rangle },$$

(34) $$R=g_{L}^{l_{R}}{\left(h_{R}^{\prime }\right)}^{r_{L}}u^{\langle l_{R},r_{L}\rangle }.$$

The round challenge is used to fold the two halves of the vectors and generators. This procedure is repeated until the vectors are reduced to final scalars $a^{*}$ and $b^{*}$. For initial vector dimension *N*, the inner-product proof contains $2\left\lceil \log_{2}N\right\rceil$ recursive group elements and two final field elements. Complete range-proof transcript contains $\left\{A,S,T_{1},T_{2},\left\{L,R\right\},\hat{t},\tau_{x},\mu ,a^{*},b^{*}\right\}$. Therefore, the proof size grows logarithmically with *N*. This statement concerns proof length; the prover and verifier still perform multi-scalar multiplications whose total work depends on *N*.

For one accessing satellite, let $V=(V_{1},\ldots ,V_{m})$ denote the vector of registered Pedersen commitments associated with its *m* quantized orbit-related attributes. These commitments remain separate public statements. The protocol aggregates their range relations by concatenating the corresponding binary witnesses into a single vector and proving one combined inner-product relation. It does not algebraically merge all commitments into one new Pedersen commitment.

(35) $$R_{\mathrm{agg}}=\left\{\left(W;w,\eta \right)|W_{k}=g^{w_{k}}h_{0}^{\eta_{k}}\wedge 0\leq w_{k}<2^{n_{k}},\forall k=1,\ldots ,q\right\}.$$

Let *q* denote the number of range values included in the proof and let $n_{k}$ be the bit length of the *k*-th range value. The total witness dimension is $N=\sum_{k=1}^{q}n_{k}$. If only non-negative *n*-bit ranges are proved, then q=m and N=mn. If both lower and upper physical bounds are enforced through Equations (27) and (28), then q=2m and, for equal bit lengths, N=2mn. The binary vector $a_{L}\in Z_{p}^{N}$ is constructed by sequentially concatenating the binary decompositions of all *q* range values. The *k*-th segment satisfies $\langle a_{L,\left[s_{k}:s_{k}+n_{k}\right]},2^{n_{k}}\rangle =w_{k}$, where $s_{k}=\sum_{r<k}^{⁠}n_{r}$. Accordingly, the vector polynomial $r\left(X\right)$ is modified as follows:

(36) $$r\left(X\right)=\left(a_{R}+z1^{N}+s_{R}X\right)\circ y^{N}+\sum_{k=1}^{q}z^{1+k}d_{k}.$$

where $d_{k}=0^{\sum_{r<k}^{⁠}n_{r}}∥2^{n_{k}}∥0^{\sum_{r>k}^{⁠}n_{r}}$. The padding vector in the *k*-th summand places $2^{n_{k}}$ in the segment corresponding to $w_{k}$ and zeros elsewhere. Each range-value commitment $W_{k}$ uses its own blinding factor $\eta_{k}$. Therefore, the blinding response becomes

(37) $$\tau_{x}=\tau_{1}x+\tau_{2}x^{2}+\sum_{k=1}^{q}z^{1+k}\eta_{k}.$$

For the two-sided interval construction, the derived blinding factors satisfy $\eta_{2j-1}=\gamma_{j}$, $\eta_{2j}=-\gamma_{j}$. The aggregation described above is performed only across the attributes of one satellite. Once generated, the same proof transcript may be sent without modification to multiple in-domain verifiers. When several satellites request access, each satellite produces its own transcript; no joint multi-prover witness is formed.

(38) $$\delta_{q,N}\left(y,z\right)=\left(z-z^{2}\right)\langle 1^{N},y^{N}\rangle -\sum_{k=1}^{q}z^{k+2}\langle 1^{n_{k}},2^{n_{k}}\rangle .$$

(39) $$g^{\hat{t}}h_{0}^{\tau_{x}}\overset{?}{=}\;g^{\delta_{q,N}\left(y,z\right)}\left(\prod_{k=1}^{q}W_{k}^{z^{k+1}}\right)T_{1}^{x}T_{2}^{x^{2}}.$$

(40) $$p=AS^{x}g^{-z1^{N}}{\left(h^{\prime }\right)}^{zy^{N}+\sum_{k=1}^{q}z^{k+1}d_{k}}h_{0}^{-\mu }.$$

Algorithm 1 shows the complete flow of non-interactive zero-knowledge proofs for multiple private input aggregation.
**Algorithm 1** Multi-Attribute Non-Interactive Zero-Knowledge Authentication**INPUT:** $g,h,g,h,\alpha ,\rho ,s_{L},s_{R},v=\left\{a,\theta ,M,\Omega \right\},ctx,L,U,\left\{v_{1},\ldots ,v_{m}\right\},\left\{\gamma_{1},\ldots ,\gamma_{m}\right\}$**OUTPUT:** {Verifier accepts, Verifier rejects}**Prover Processes:**$\mathrm{Check}\;\mathrm{that}\;\mathrm{each}\;\mathrm{registered}\;\mathrm{commitment}\;\mathrm{satisfies}\;V_{j}=g^{\upsilon_{j}}h_{0}^{\gamma_{j}}$$\mathrm{For}\;\mathrm{each}\;\mathrm{attribute}\;j,\;\mathrm{derive}\;\mathrm{the}\;\mathrm{bound}\;\mathrm{commitments}\;W_{j,-}=V_{j}g^{-L_{j}},\;W_{j,+}=g^{U_{j}}V_{j}^{-1}$$\mathrm{Define}\;\mathrm{the}\;\mathrm{corresponding}\;\mathrm{range}\;\mathrm{values}\;w_{j,-}=\upsilon_{j}-L_{j},\;w_{j,+}=U_{j}-\upsilon_{j}$$\mathrm{Concatenate}\;\mathrm{binary}\;\mathrm{decompositions}\;\mathrm{into}\;a_{L}\in Z_{p}^{N}$$\mathrm{Set}\;a_{R}=a_{L}-1^{n}$$\mathrm{Sample}\;\alpha ,\rho ,\tau_{1},\tau_{2}\leftarrow Z_{p},\;s_{L},s_{R}\leftarrow Z_{p}^{N}$ComputeAandSusing Equations (13) and (14)Deriveyandzfromctx,AandS$\mathrm{Construct}\;\;l\left(X\right),r\left(X\right)\;\mathrm{and}\;\;t\left(X\right)=\langle l(X),r(X)\rangle \;\mathrm{using}$ the aggregated relation in Equation (18)$\mathrm{Commit}\;\mathrm{to}\;t_{1}\;\mathrm{and}\;t_{2}:T_{1}=g^{t_{1}}h_{0}^{\tau_{1}},T_{2}=g^{t_{2}}h_{0}^{\tau_{2}}$Derivexfromthecompletetranscript$\mathrm{Compute}\;l=l\left(X\right),\;r=r\left(X\right),\;\hat{t}=t\left(X\right),\;\mu =\alpha +\rho x\;\mathrm{and}\;\tau_{x}=\tau_{1}x+\tau_{2}x^{2}+\sum_{k=1}^{q}z^{1+k}\eta_{k}$$\mathrm{Construct}\;P_{\mathrm{IP}}\;\mathrm{according}\;\mathrm{to}$ Equation (26)
Execute the recursive inner-product argument    Foreacharoundr         Splitthecurrentvectorsandgenerators         ComputeLandR         Derivex         Foldthevectors,genetatorsandcommitment    $\mathrm{Output}\;\mathrm{the}\;\mathrm{final}\;\mathrm{scalars}\;a^{*}\;\mathrm{and}\;b^{*}$$\mathrm{Output}\;\pi =\left\{A,S,T_{1},T_{2},\left\{L,R\right\},\hat{t},\tau_{x},\mu ,a^{*},b^{*}\right\}$**Verifier Processes:**CheckthevalidityofepochandsidRejectaduplicatedsidthathasalreadybeenacceptedRecomputeallFiat–Shamirchallengesy,z,xandtheinner-productchallengesVerifytheaggregatedpolynomial-consistencyequationusing Equation (39)
$\mathrm{Reconstruct}\;P_{\mathrm{IP}}\;\mathrm{and}\;\mathrm{verify}\;\mathrm{the}\;\mathrm{recursive}\;\mathrm{inner}-\mathrm{product}\;\mathrm{proof}\;\mathrm{using}\;\left\{L,R\right\},a^{*},b^{*}$Ifallequationshold,recordsidandreturnAccept;otherwisereturnReject

### 3.2. Cross-Domain Collaborative Satellite Resource Allocation

After a satellite successfully completes the zero-knowledge authentication procedure, the domain controller registers it as an available resource provider and updates the virtual resource pool. Satellites failing authentication are excluded from scheduling, while satellites awaiting verification remain unavailable until the next scheduling epoch. Consequently, every scheduling decision is performed over the authenticated resource set, ensuring that resource allocation is both secure and consistent with the current network membership.

The LEO satellite network consists of *M* domains with *N* LEO satellites in each domain. The number of tasks to be processed varies from one domain to another, and this spatio-temporal non-uniformity in task distribution results in certain domains having resource reserves that cannot satisfy the full demand of tasks to be processed. Domains that can fully allocate the local domain task resources and have resource surplus are considered idle domains, denoted as $C_{idle}=\left\{C_{i_{1}},\ldots ,C_{i_{\phi }}\right\}$, and on the contrary, domains that cannot fully execute domain tasks are considered as busy domains, denoted as $C_{busy}=\left\{C_{b_{1}},\ldots ,C_{b_{\varphi }}\right\}$, where ϕ+φ=M and $C_{idle}\cup C_{busy}=C$. In order to balance the load difference of task processing among different domains, an actor-critic offline reinforcement learning scheme is first used within domains to maximize the number of domain-local task completions, assisted by an inter-domain early-exit matching game scheme that matches a suitable $C_{idle}$ to $C_{busy}$ to share the resource pressure of unfinished tasks.

Satellite communication networks must perform various tasks with different functions; we classify tasks as latency-sensitive, computation-intensive, and storage-intensive according to the required resources. There are differences in the type and size of resources required to fulfill different tasks. Under the supervision of the ground control center, a high-priority type of task exists that needs to be executed instantly and should be given priority in the resource allocation process to obtain the required resources. We model the satellite network multidimensional resource multi-task resource matching problem as an actor-critic reinforcement learning process in a cooperative environment, where the problem decision process is a Markov decision process. The actor policy network generates the resource allocation action $a_{t}$ by receiving the current state $s_{t}$ and generating the resource allocation action $a_{t}$; the Critic policy network is responsible for estimating the value of performing the action $a_{t}$, $Q\left(s_{t},a_{t}\right)$, in conjunction with the current network state $s_{t}$. The resource allocation scheme for domain $C_{i}$ is generated by the domain head of this domain acting as an intelligent and performing a reinforcement learning process. The remaining resources of the *N* members of this domain constitute a virtual resource pool, and multidimensional resources are often represented using resource cubes, which we consider to be classified as computational resources $R_{C}$, storage resources $R_{S}$, and transmission resources $R_{T}$. The available resources of domain $C_{i}$ in the *t*-th time slice are denoted as

(41) $$R_{i}^{t}=\left\{R_{i_{C}}^{t},R_{i_{S}}^{t},R_{i_{T}}^{t}\right\},\;i\in \left\{1,\ldots ,M\right\},$$

where $V\left(R_{i_{C}}^{t}\right)$, $V\left(R_{i_{S}}^{t}\right)$, and $V\left(R_{i_{T}}^{t}\right)$ are denoted as the remaining amount of computational, storage, and transmission resources in domain $C_{i}$ in the *t*-th time slice, and the function $V\left(\cdot \right)$ is used to compute the amount of the currently allocable resources. Of course, $0\leq V\left(R_{i_{C}}^{t}\right)\leq V\left(R_{i_{C}}\right)$, but more importantly, the resources of the satellite member ${CM}_{ij}$ in domain $C_{i}$ at the *t*-th time slice are denoted as $R_{ij_{\zeta }}^{t}$ and satisfy

(42) $$V\left(R_{i_{\zeta }}\right)=\sum_{j\in \left\{1,\ldots ,N\right\}}V\left(R_{ij_{\zeta }}\right),i\in \left\{1,\ldots ,M\right\},\zeta \in \left\{C,S,T\right\},$$

(43) $$V\left(R_{ij_{C}}^{t}\right)\geq V\left(R_{ij_{C}}^{t+1}\right)\geq 0,i\in \left\{1,\ldots ,M\right\},j\in \left\{1,\ldots ,N\right\},\forall t\in \left\{1,\ldots ,T-1\right\}.$$

Different tasks have different priorities in the execution order. The execution priority of task $T_{ir}$ in domain $C_{i}$ can be expressed as $T_{ir,P}^{t}$ with the following equation:

(44) $$T_{ir,P}^{t}=\left\{\begin{array}{c} 0,if\;\exists \zeta \;V\left(T_{ir,R_{\zeta }}^{t}\right)>V\left(R_{i_{\zeta }}^{t}\right),\zeta \in \left\{C,S,T\right\}\\ \omega_{1}{\cdot g\left(T_{ir}\right)+\omega }_{2}\cdot e^{h\left(T_{ir}\right)}{+\omega }_{3}\cdot r\left(T_{ir}\right),else \end{array}\right.,$$

where $\omega_{1}+\omega_{2}+\omega_{3}=1$ and $r\in \left\{1,\ldots ,R\right\}$. If the resources required by task $T_{ir}$ are no longer available for complete allocation, then it directly does not participate in the resource allocation process; in addition, the priority of task $T_{ir}$, $T_{ir,P}^{t}$, is jointly affected by the task type, the resource matching degree, and the task submission time. The function $g\left(\cdot \right)$ is a step function related to the type of task $T_{ir}$, and the immediate task of the ground control center has the highest priority. Although the divergence itself is non-negative, the matching score *h* is obtained through a signed mapping of the distance. Function $h\left(\cdot \right)$ is a Kullback-Leibler function related to the degree of resource matching of the task $T_{ir}$, which is related with

(45) $$D_{KL}\left(T_{ir,R}^{t}\|R_{i}^{t}\right)=V\left(T_{ir,R_{C}}^{t}\right)\log\frac{V\left(T_{ir,R_{C}}^{t}\right)}{V\left(R_{i_{C}}^{t}\right)}+V\left(T_{ir,R_{S}}^{t}\right)\log\frac{V\left(T_{ir,R_{S}}^{t}\right)}{V\left(R_{i_{S}}^{t}\right)}+V\left(T_{ir,R_{T}}^{t}\right)\log\frac{V\left(T_{ir,R_{T}}^{t}\right)}{V\left(R_{i_{T}}^{t}\right)}.$$

The resource vectors are first normalized before calculating the matching measure, such that each resource vector represents the relative distribution of different resource dimensions rather than the absolute resource amount. Specifically, the required resource vector and available resource vector are transformed into normalized resource distributions. The function $r\left(\cdot \right)$ is a sigmoid function related to the submission time $t_{ir}^{com}$ of the task $T_{ir}$, where the earlier the submission at the same time has a higher task priority, with the following expression:

(46) $$r\left(T_{ir}\right)=\frac{1}{1+e^{\varepsilon \left(t_{ir}^{com}-t^{cur}\right)}}\;,\;\varepsilon >0.$$

Obviously, the setting of 0<ε<1 slows down the growth rate of task execution priority over time.

The satellite resource allocation action for domain $C_{i}$ in the *t*-th time slice is the process of finding a resource block matching its requirement for the appropriate task $T_{ir}$ from the existing remaining resources $R_{i}^{t}$ in the current virtual resource pool. The inter-satellite communication is constantly changing, and considering the limitations of the on-board transponders, we assume that only one task is allocated its required resources within the same time slice. The intra-domain resource allocation satisfies the following restrictions:

(47) $$\sum_{t\in \left\{1,\ldots ,T\right\}}^{⁠}\sigma_{i,r}^{t}=1,$$

(48) $$\sigma_{i,r}^{t}\cdot x_{ir,R_{\zeta }}^{t}=V\left(R_{i_{\zeta }}^{t}\right)-V\left(R_{i_{\zeta }}^{t+1}\right),\zeta \in \left\{C,S,T\right\},$$

(49) $$V\left(T_{ir,R_{\zeta }}^{t}\right)\leq \sum \sigma_{i,r}^{t}\cdot x_{ir,R_{\zeta }}^{t},\zeta \in \left\{C,S,T\right\},$$

where $\sigma_{i,r}^{t}\in \left\{0,1\right\}$ denotes whether domain $C_{i}$ allocates resources for task $T_{ir}$ at the *t*-th time slice, and $\left\{x_{ir,R_{\zeta }}^{t}|\zeta \in \left\{C,S,T\right\}\right\}$ is the amount of various resources allocated by domain $C_{i}$ for task $T_{ir}$ at the *t*-th time slice.

Considering the state of the satellite resource and the task demand, we portray the decision to allocate the satellite resource as a state-action-return-driven MDP process. The state space $S_{i}^{t}$ of domain $C_{i}$ at time slice *t* can be expressed as

(50) $$S_{i}^{t}=\left\{R_{i}^{t},T_{i}^{t}\right\}=\left\{\left\{R_{i_{C}}^{t},R_{i_{S}}^{t},R_{i_{T}}^{t}\right\},\left\{T_{ir,P}^{t},T_{ir,R}^{t}\right\}\right\}.$$

The resource state $R_{i}^{t}=\left\{R_{i_{C}}^{t},R_{i_{S}}^{t},R_{i_{T}}^{t}\right\}$ describes the currently available computation, storage, and transmission resources in domain $C_{i}$. The task state $T_{i}^{t}$ describes the set of pending tasks waiting for resource allocation, where each task $T_{ir}$ is characterized by its priority $T_{ir,P}^{t}$ and resource requirement vector $T_{ir,R}^{t}$. The decision to allocate resources to domain tasks constitutes the action space of domain $C_{i}$, which can be represented as

(51) $$A_{i}^{t}=\left\{T_{ir},x_{ir,R_{\zeta }}^{t}\right\}=\left\{T_{ir},\left\{x_{ir,R_{C}}^{t},x_{ir,R_{S}}^{t},x_{ir,R_{T}}^{t}\right\}\right\}.$$

Resource allocation is based on the unit of resource blocks, and matching the most appropriate resource blocks for a task helps reduce resource fragmentation and avoid interplanetary communication brought about by the joint execution of tasks by multi-satellite equipment.

For satellite communication networks, the goal of resource allocation for each domain is to maximize the task local completion rate, i.e., to reduce unnecessary inter-satellite collaboration, which brings severe instability to dynamic narrowband satellite communications. However, different tasks require different types and numbers of resources; maximizing the number of completed tasks is the optimization objective. At the same time, due to the variability of task attributes, higher-priority tasks need to obtain resource allocation faster, where delay-sensitive tasks and possible temporary instantaneous commands issued by the ground control center are involved. Unlike the standard resource allocation unit in virtual resource pools, we consider the resources provided by the same satellite equipment, called resource block. Resource blocks that match the resource requirements of a mission bring a higher overall value when they receive an allocation. The value generated by a resource allocation action is represented using the reward function $R_{i}$, denoted as

(52) $$R_{i}=\left\{\begin{array}{c} 1-\vartheta_{OD}\cdot \left(e^{D_{KL}\left(x_{ir,R_{\zeta }}^{t}\|T_{ir,R_{\zeta }}^{t}\right)}-1\right),\forall r,\zeta \;D_{KL}\left(\cdot \right)\neq 0,\forall \zeta \;V\left(T_{ir,R_{\zeta }}^{t}\right)\leq x_{ir,R_{\zeta }}^{t}\\ 1,\forall r,\zeta \;D_{KL}\left(\cdot \right)=0\\ -\vartheta_{PD}\cdot e^{D_{KL}\left(T_{ir,R_{\zeta }}^{t}\|x_{ir,R_{\zeta }}^{t}\right)},\forall r,\zeta \;D_{KL}\left(\cdot \right)\neq 0,\exists \zeta \;V\left(T_{ir,R_{\zeta }}^{t}\right)>x_{ir,R_{\zeta }}^{t} \end{array}\right.,$$

where $\vartheta_{OD}\in \left(1,10\right)$ and $\vartheta_{PD}\in \left(0,1\right)$. Rewards get penalized accordingly when resources are over-allocated, and performing resource allocation actions should be avoided when the remaining resources of the domain are not enough for a task to get a full resource allocation.

The time difference method is used to optimize the critic parameters of the loss function to update the critic network. The formula for updating the parameters of the critic network is as follows:

(53) $$J\left(\theta^{i}\right)=E_{\left(s,a,r,\tilde{s}\right)∼D}\left[{\left(Q_{\theta^{i}}\left(s,a\right)-y_{i}\right)}^{2}\right],$$

(54) $$y_{i}=R_{i}+\varrho \min E_{\tilde{a}∼D\left(\tilde{s}\right)}\left[Q_{\theta^{i}}^{\iota }\left(\tilde{s},\tilde{a}\right)\right],\iota \in \left\{1,2\right\},$$

where $\left(s,a,r,\tilde{s}\right)$ is the empirical data, *D* is an offline dataset composed of scheduling records generated from existing cross-domain resource managers and hybrid heuristic strategies, and $Q_{\theta^{i}}\left(s,a\right)$ denotes the current Q network estimate for domain $C_{i}$. However, in offline reinforcement learning, the critic policy network in state $S_{i}^{t}$ is prone to over-value actions that have not appeared in the offline experience pool, and we use conservative Q-learning to constrain the valuation of the unknown actions with the following formula:

(55) $$J_{CQL}\left(\theta^{i}\right)=J\left(\theta^{i}\right)+\rho \left(E_{a∼\pi \left(s\right)}\left[Q_{\theta^{i}}\left(s,a\right)\right]\right)-E_{a∼D\left(s\right)}\left[Q_{\theta^{i}}\left(s,a\right)\right],$$

where $\pi \left(s\right)$ is an out-of-strategy distribution and $\pi \left(s\right)\cap D\left(s\right)=\emptyset$. When updating the parameters of the actor-network, the critic network generates action estimates based on the actions sampled by the actor-network. The critic is updated using the policy gradient method based on the estimates. The parameters of the critic network are updated in the following way:

(56) $$\nabla_{\theta }J_{CQL}\left(\theta^{i}\right)=E_{\left(s,a,r,\tilde{s}\right)∼D}\left[\nabla_{\theta }Q_{\theta^{i}}\left(s,a\right)\left(Q_{\theta^{i}}\left(s,a\right)-y_{i}\right)\right]+\rho \nabla_{\theta }\left(E_{a∼\pi \left(s\right)}\left[Q_{\theta^{i}}\left(s,a\right)\right]-E_{a∼D\left(s\right)}\left[Q_{\theta^{i}}\left(s,a\right)\right]\right).$$

The complete training process is shown in Algorithm 2.
**Algorithm 2** Offline Reinforcement Learning Training**INPUT:** replay buffer *D*, actor network $A_{\phi }$, critic network $Q_{\theta }$, $\omega_{1}$, $\omega_{2}$, $\omega_{3}$, ε, $\vartheta_{OD}$,$\vartheta_{PD}$, ϱ, ρ, state space *S*, action space *A***OUTPUT:** actor network $A_{\phi }$, critic network $Q_{\theta }$Initialize replay buffer *D*, $A_{\phi^{i}}$, $Q_{\theta^{i}}$For Iteration = 1, 2,..., L **do**     Initialize member ${CM}_{ij}$ state space $S_{i}$ action space $A_{i}$     For t = 1, 2,..., T do          While exist tasks that have not been assigned              The state $S_{i}^{t}$ of domain $S_{i}$ is sent to the actor              Generate $A_{i}^{t}∼D\left(\cdot |S_{i}^{t}\right)$ or new action $A_{i}^{t}∼\pi \left(\cdot |S_{i}^{t}\right)$              Calculate the instant reward $R_{i}$ using Equation (52)              Calculate action estimated value $Q_{\theta_{i}}\left(s,a\right)$ using Equation (53)              Update the resource state space $R_{i}^{t}$ of the domain $C_{i}$              Update the task state space $T_{i}^{t}$ of the domain $C_{i}$              Store $\left\{S_{i}^{t},A_{i}^{t},R_{i},S_{i}^{t+1}\right\}$ of each step into buffer *D*              Calculate the loss function $J_{CQL}\left(\theta^{i}\right)$              Optimize critic network using Equation (56)          Count tasks that have fully allocated resources     End ForEnd For

The execution demand of tasks with unallocated resources within domains is satisfied through collaborative resource allocation between domains. Busy domain $C_{busy}$ and idle domain $C_{idle}$ are regarded as two agents, respectively; in particular, $C_{busy}$ acts as a demander while $C_{idle}$ acts as a provider. Based on the task classification of busy domain $C_{busy}$ and the virtual resource pool of idle domain $C_{busy}$, we construct a preference list to express their expectations, which describes the priority matching relationship of different domains. The function $M\left(\cdot \right)$ denotes a one-to-one matching relationship, i.e., $M\left(C_{idle}^{\phi }\right)\in C_{busy}$, and it is worth noting that $M\left(C_{idle}^{\phi }\right)=C_{busy}^{\varphi }$ when and only when $M\left(C_{busy}^{\varphi }\right)=C_{idle}^{\phi }$. After intra-domain resource allocation, some tasks may remain unserved because the local virtual resource pool is insufficient. Cross-domain resource sharing is therefore performed between busy domains and idle domains. A busy domain acts as a resource demander, whereas an idle domain acts as a resource provider. Each busy domain $C_{busy}^{i}$ constructs an individual preference list over candidate idle domains according to transmission distance. Idle domains that exceed the admissible distance threshold or cannot satisfy the required resource vector are regarded as unacceptable candidates. In contrast, all idle domains rank busy domains using the same task-priority order $P\left(C_{idle}^{1}\right)=P\left(C_{idle}^{2}\right)=\cdots =P\left(C_{idle}^{\phi }\right)$. Specifically, the busy domains are processed in descending order of their task priorities $T_{i,p}$. For each busy domain, the algorithm scans its distance-based preference list and selects the first currently unmatched idle domain that satisfies the resource and distance constraints. Once an assignment is established, the selected idle domain is removed from the available set. If no feasible idle domain is found, the busy domain remains unmatched during the current time slot. This procedure produces resource-feasible one-to-one assignments, ensures that higher-priority busy domains are processed before lower-priority ones, and terminates after a finite number of candidate checks. The specific bilateral early-exit matching game algorithm is shown in Algorithm 3.
**Algorithm 3** Bilateral early-exit Matching Game**INPUT:** busy domain set $C_{busy}$, idle set $C_{idle}$, task priority $T_{i,p}$, resource required $R_{i}^{busy}$, resource remaining $R_{j}^{idle}$, inter-domain distance threshold $d_{max}$**OUTPUT:** cross domain match result *M*Initialize M←∅ and $U_{idle}\leftarrow C_{idle}$For each busy domain $C_{i}^{busy}$     Construct $P_{i}^{busy}$ by sorting idle domains in ascending order of distanceRemove from $P_{i}^{busy}$ the idle domains whose transmission distances exceed $d_{max}$Sort the busy domains in descending order of task priority $T_{i,p}$Break equal-priority ties deterministically using the domain indexFor each busy domain $C_{i}^{busy}$ in the sorted busy-domain set do    For each idle domain $C_{j}^{idle}$ in $P_{i}^{busy}$ do         If $C_{j}^{idle}\in U_{idle}$ and $R_{j}^{idle}$ satisfies $R_{i}^{busy}$ then             $M\leftarrow M\cup \left\{(C_{i}^{busy},C_{j}^{idle})\right\}$             $U_{idle}\leftarrow U_{idle}\setminus \left\{C_{j}^{idle}\right\}$             Break         End if    End forEnd forReturn *M*

## 4. Theoretical Analysis

### 4.1. Analysis of Protocol Robustness

Compared with link continuity, the aggregated non-interactive zero-knowledge authentication protocol mainly relies on single-packet reachability. Objectively, LEO inter-satellite links are difficult to avoid the possibility of narrowband, time-varying and intermittent interruptions. The one-time proof transmission of the non-interactive design replaces the multi-round interactive authentication sessions, which suppresses the problems of link retries and state rollbacks caused by fluctuating links. Verifier no longer needs to continuously maintain the session state and issue online challenges, but instead seeks independent judgments through local verification.

As satellites move at high speed, the topology of low-orbit satellite networks is constantly changing, and the verifier set used for access verification is also adjusted along with the satellite orbits and node rotation. The verification logic of the proposed authentication protocol is not strictly bound to the stable adjacency relationship of the satellite applying for network access, nor does it require the designation of specific satellites to participate in the verification.

The accuracy of external devices in measuring and estimating orbital parameters is limited by measurement noise and short-term disturbances. However, the estimated orbital parameters do not directly impact the accuracy of the authentication protocol. The actual data used to generate the proof is the high-precision orbital state maintained internally within the constellation, rather than external orbital estimates. The verifier does not directly access the raw declaration data but instead assesses the legitimacy of the satellite’s identity by analyzing the correctness of the proof, which encapsulates the orbital state within strict constrained intervals.

The security of the proposed authentication protocol is based on the hardness of the discrete logarithm problem over the selected elliptic curve group. Furthermore, the Fiat–Shamir transform is analyzed in the Random Oracle Model, where the hash function $RO\left(\cdot \right)$ is modeled as a random oracle. Since the challenge is deterministically derived from the public transcript through SHA-256, neither the prover nor any adversary can choose the challenge adaptively after observing the commitments, thereby preserving the soundness guaranteed by the Fiat–Shamir transform.

### 4.2. Analysis of Protocol Security

This section provides a formal description of the security model for the proposed non-interactive authentication protocol. The satellite seeking access serves as the Prover, while the in-domain satellite functions as the Verifier. Under the Dolev-Yao communication adversary model, the following types of adversaries are considered:

1. Passive adversary: Attempts to derive internal orbital parameters or other valuable information from the proof.
2. Active adversary: Impersonates and replays historical proofs or forges proofs without possessing legitimate orbital parameters.
3. Colluding adversaries: Multiple malicious entities collaborate to share observed proofs in order to enhance their forgery success rate.
4. Curious verifier: Executes the verification process but attempts to extract internal orbital parameters from the proof.

The overall security is derived from the underlying scope structure. When the model assumptions hold true and the Fiat–Shamir heuristic is applied within the random oracle model, properties such as completeness, soundness, and replay resistance are ensured. The following proof sketch elucidates the key mechanisms and intuitions underlying each security attribute.

1. Completeness: Assuming the Prover possesses a valid witness and adheres to the protocol by constructing commitments and proofs accordingly, the Verifier will conduct the prescribed equation checks and approve the proof. Intuitively, completeness stems from two factors: first, the scope constraints are satisfied with an honest witness υ; second, the relationships between commitments and polynomials within the proof are self-consistent as per the construction. The only scenario in which an honest proof might be erroneously rejected is in the event of a hash collision.
2. Soundness: The equation checks performed by the Verifier collectively enforce consistency among commitments, vector relationships, and polynomial inner product relationships. Any successful forgery will inevitably lead to one of the following contradictions. Within the random oracle model, the challenge value is determined by the commitment, preventing the adversary from retroactively selecting a challenge to match forged data. Successfully passing the equation checks would violate the soundness of the underlying inner product argument, contradicting standard security conclusions. Alternatively, to satisfy the verification equation, the adversary would need to provide contradictory openings under the same commitment, which is infeasible when commitment binding holds. Therefore, under the assumptions of the discrete logarithm problem and the random oracle model, the probability of an adversary forging a verifiable proof without knowledge of the valid witness υ is negligible. We emphasize that the proposed proof establishes the correctness of a previously registered orbit-information commitment and its associated range constraints. Authorization of a satellite is assumed to be guaranteed by the prior registration procedure, whereas the zero-knowledge proof is responsible only for proving possession of the registered witness without revealing it.
3. Replay Resistance: Two replay scenarios are addressed. For cross-domain replay, an adversary may transcribe and transfer a captured proof to another domain; however, the algebraic consistency of the original proof will be disrupted due to domain-specific variations in the challenge value. Regarding replay across time windows, the epoch is incorporated into challenge derivation, resulting in altered challenge values for each new time window and rendering the original proof invalid. It is important to note that while an adversary may not be able to distinguish between identical proofs replayed within the same domain and time window, zero-knowledge proofs themselves are not expected to provide uniqueness detection.
4. Collusion Resistance: Collusion does not significantly enhance forgery capabilities. All satellites within the same domain and epoch receive identical aggregated proofs from the Prover. Since all colluding adversaries observe exactly the same public transcript while none possesses the registered commitment opening, exchanging observed proofs does not provide additional information that increases the probability of constructing a valid forgery beyond that of a single adversary.

The proposed authentication proof differs from standard Bulletproofs mainly in two aspects: orbit-information binding and multi-parameter aggregation. Let the orbital parameter tuple be $\upsilon =\left\{a,\theta ,M,\Omega \right\}$, and let each parameter $\upsilon_{j}$ be committed as $V_{j}=g^{\upsilon_{j}}h^{\gamma_{j}}$, where $\gamma_{j}$ is an independent blinding factor. The public statement proves that each committed orbital parameter lies within its legal interval and that the aggregated binary decomposition is consistent with the committed values. The soundness of the range relation follows from the standard Bulletproofs inner-product argument. If an adversary generates an accepting proof for an invalid orbital parameter, then either the range-proof relation is violated, contradicting the soundness of Bulletproofs, or the adversary opens the same Pedersen commitment to two different values, which breaks the computational binding of Pedersen commitments under the discrete logarithm assumption. The aggregation process only concatenates multiple *n*-bit witnesses into an mn-dimensional vector relation and modifies the corresponding polynomial and verification equations accordingly. Therefore, an invalid component cannot be hidden inside the aggregated proof, since any inconsistency changes the final inner-product and polynomial verification equations.

The Fiat–Shamir challenges are derived from the commitments and the public session context, while the actual orbital parameters are protected by independent Pedersen blinding factors. A simulator can sample random response values and program the random oracle outputs to generate a transcript satisfying the same verification equations as a real proof. Since the simulated transcript has the same distribution as the real transcript and does not require knowledge of υ, the verifier learns no information about the committed orbital parameters beyond the fact that they satisfy the declared range constraints. Therefore, the orbit-information binding and aggregation operations preserve the zero-knowledge property of the underlying bulletproof construction.

## 5. Experimental Analysis

In this section, we first describe the system model parameters and algorithm parameters involved in the simulation experiments. Then, we verify the validity of the proposed scheme and analyze its performance.

### 5.1. Experimental Setup

In the simulation, we construct a simulated LEO satellite constellation; specifically, the satellite constellation has an orbital altitude of 750 km, and 300 LEO satellites are uniformly deployed in 20 satellite orbits with an inclination angle of 30°. In this paper, we do not consider the uplink channel of the ground control station or ground-based IoT and only consider the inter-satellite narrowband transmission of the non-terrestrial network, with the inter-satellite transmission narrowband bandwidth of 50 kHz and the narrowband data transmission rate of 15 kbps. All satellites are divided into 10 domains according to the longitude and latitude in the space where the satellites are located, and each domain randomly selects a domain head. Domain heads are regarded as intelligences with dynamic resource allocation capability, and in the offline learning phase, the learning rate is set to $1\times 10^{-3}$ with a discount factor of 0.99. In addition, the offline training set is 20,000. The offline dataset is generated by greedy and heuristic schedulers to provide diverse state-action trajectories. These schedulers are regarded as behavior policies rather than optimal policies. The proposed RL agent further improves the decision-making policy by maximizing the long-term reward, and thus its performance is not limited by the quality of the behavior policies.

The proposed intra-domain resource allocation algorithm adopts a standard actor-critic offline reinforcement learning framework. Both the actor and critic are implemented as fully connected neural networks with three hidden layers of 256 neurons using the ReLU activation function. During offline training, mini-batches of 128 samples are uniformly drawn from the replay buffer. The conservative regularization coefficient in Equation (55) is set to 0.1. Each experiment is trained for 5000 epochs, and the model with the highest average validation reward is selected for evaluation. To ensure a fair comparison, all baseline methods were re-implemented and evaluated under the same satellite constellation, communication model, task set, and resource constraints as the proposed method.

### 5.2. Authentication and Communication Overhead

This section analyses the performance of the proposed authentication scheme by comparing different authentication schemes. In this section, we consider the inter-satellite communication process for intra-domain authentication and test it with a PC configured with Intel Core i9 and RAM = 32 GB instead of a LEO satellite. On the terminal, we have implemented and averaged the operations of hashing, scale multiplication, and symmetric encryption required for authentication using the OpenSSL library, respectively. The results are recorded in Table 1.

In this paper, we have analyzed the proof and verification process for authentication on different satellites and recorded the overall computational overhead, which is shown in Table 2. The results show that the proposed scheme in this paper has a high computational overhead in generating authentication evidence and the verification process. However, the analysis reveals that the computational overhead of the process is mainly concentrated in the verification evidence generation process. Within the same domain and epoch, the same proof transcript can be forwarded without modification to multiple authorized verifiers, which independently execute the verification algorithm. The authentication computational overhead of the proposed scheme produces a certain degree of reduction as the number of authentication devices increases. Speculatively, when the number of authenticated entities in the domain reaches 10, the computational overhead can be reduced by 23.7% compared to independent zero-knowledge authentication. The reported reduction is achieved because the four orbital attributes are aggregated into a single Bulletproof instance. Compared with independently generating and verifying four range proofs, the proposed construction reduces redundant proof components and verifier computations while maintaining the same security guarantees.

Further, we have analyzed the communication overheads of the authentication policies and recorded the overheads of the relevant actions involved in the authentication process, as shown in Table 3. Table 4 describes in detail the interactive communication overheads of different authentication strategies. The communication overhead is analytically derived from the cryptographic operations involved in each scheme. This paper’s proposed scheme applies to LEO satellite narrowband communication scenarios, and the communication overhead between the authenticating parties is only 38.6% of the overhead of the symmetric encryption-based authentication strategy proposed by Liu et al. [10]. The non-interactive authentication scheme significantly reduces the device communication overhead. However, the scheme imposes requirements on the computational power of the satellite to be authenticated, as it undertakes more authentication evidence computation operations.

Table 5 presents the end-to-end authentication delay under different numbers of authenticated satellites. Although the proposed scheme introduces higher computation overhead due to the zero-knowledge proof generation, the communication overhead is significantly reduced compared with existing schemes. The proposed scheme does not achieve the lowest delay when only one satellite is authenticated, because the computation cost dominates the total delay. However, with the increase in authenticated satellites, the batch authentication mechanism effectively amortizes the computation overhead and avoids repeated transmission of authentication messages. Therefore, the proposed scheme achieves lower end-to-end authentication delay in large-scale satellite access scenarios. This result demonstrates that the communication-computation trade-off of the proposed scheme is suitable for narrowband LEO satellite networks with frequent multi-satellite authentication requirements.

### 5.3. Analysis of Resource Allocation Strategies

**Figure 3:**
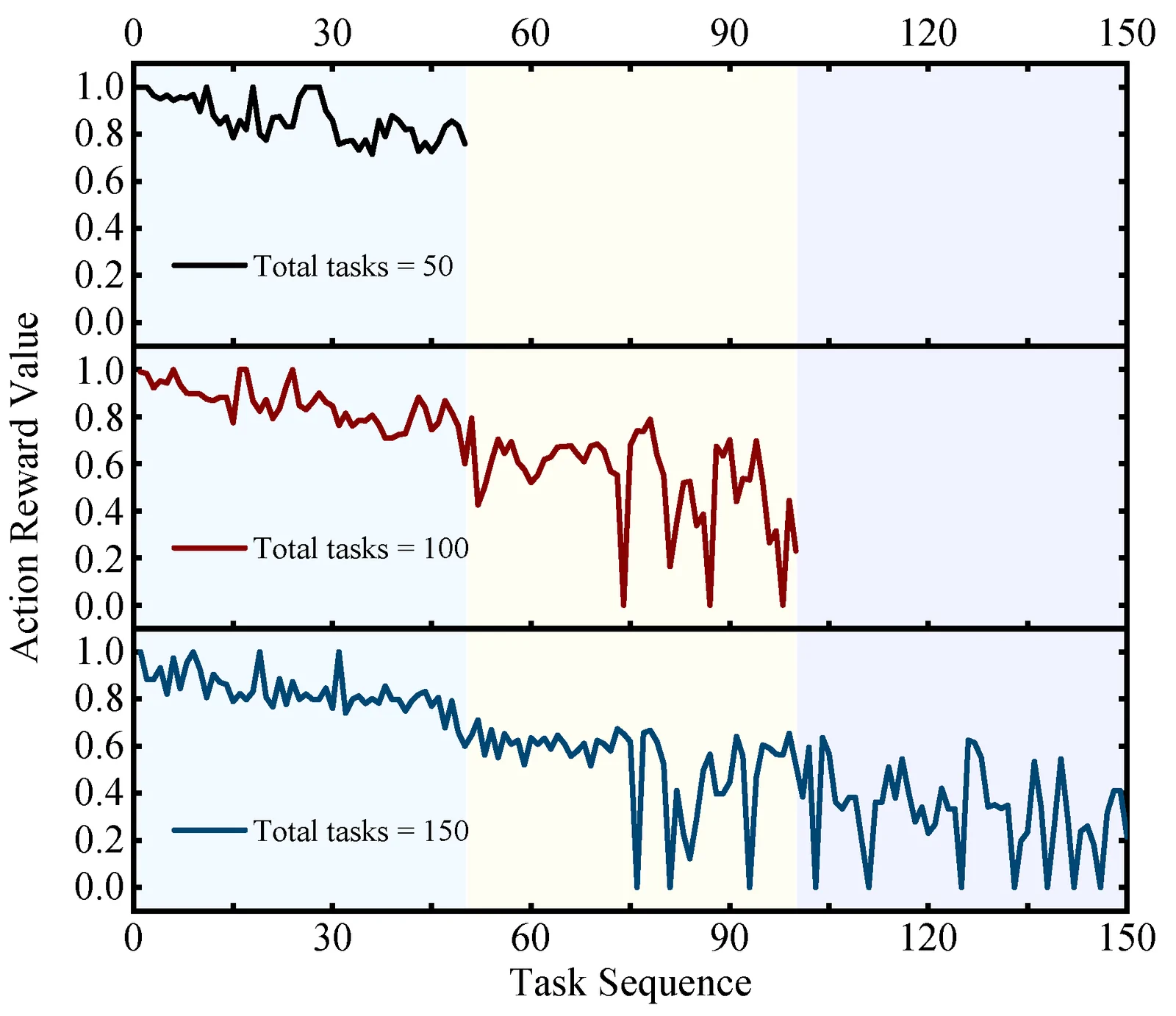
Reliability analysis of resource allocation strategies.

The proposed strategy includes an intra-domain resource allocation scheme and inter-domain task collaboration. First, we analyze the intra-domain resource allocation strategy based on offline reinforcement learning. Figure 3 records the action reward values of resource allocation operations for each task with different numbers of tasks. The analysis shows that for dynamic task resource allocation, a relatively excellent resource allocation scheme can be selected when the first arriving task carries out resource allocation, which is mainly manifested in the higher reward value of the action. As more satellite resources are allocated, there will be fewer resource allocation schemes to choose from when resource allocation is performed for subsequent tasks because factors such as task occupancy, resource wastage, or even difficulty meeting the resource requirements for task execution will intensify. The number of tasks with unsuccessful resource allocation will increase rapidly with the increase in task sequences. In order to prevent the situation where some resources are allocated but the resource demand for task execution is not met, we set the limitations related to reward penalty points, so the action reward value of the tasks that are directly skipped without allocating resources is set to 0.

**Figure 4:**
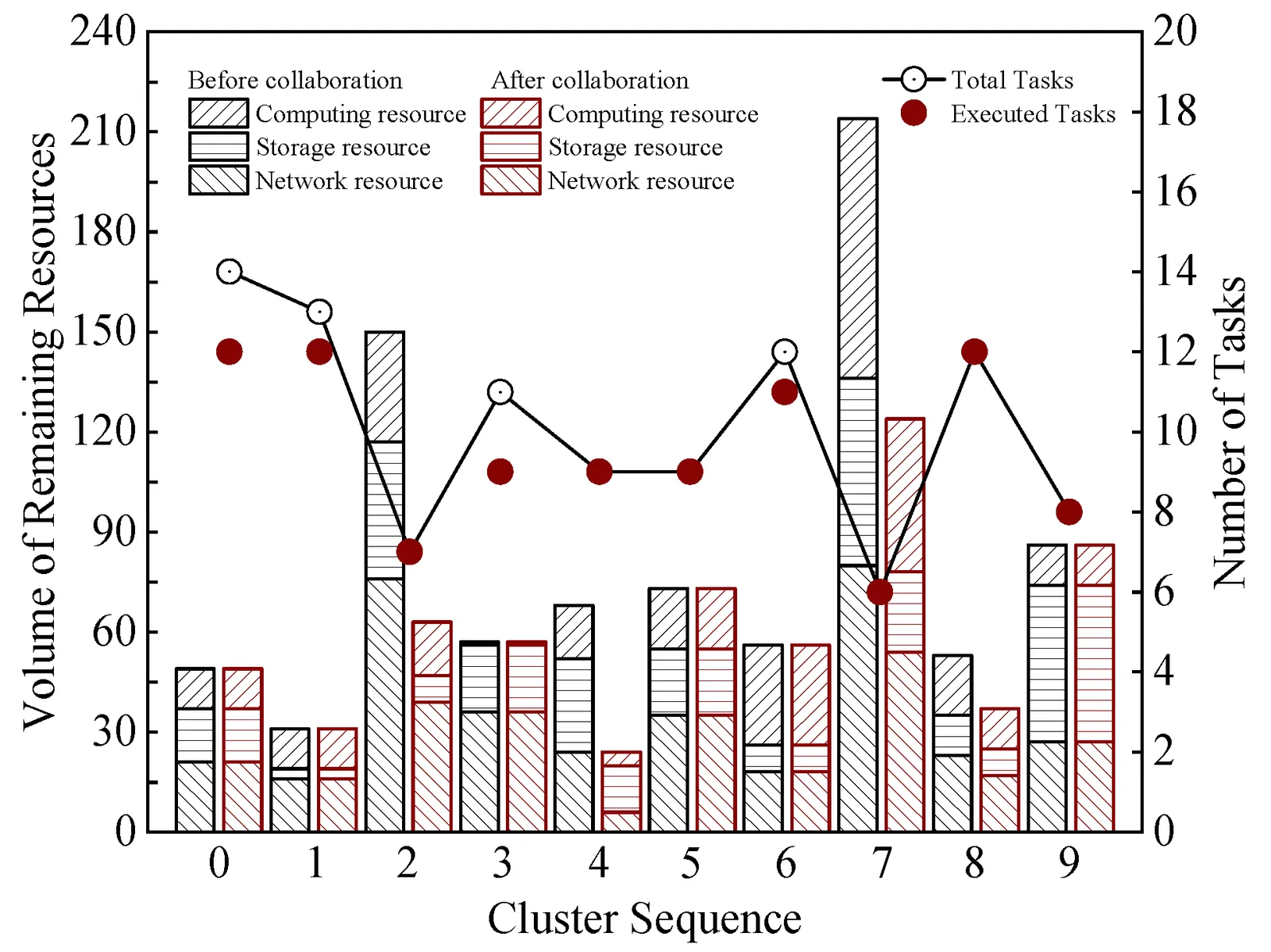
Record of variability in resource allocation for tasks in different domains.

In the absence of optimal inter-domain task scheduling, the number of task executions between different domains varies to some extent due to task allocation’s temporal and spatial variability. Figure 4 records the differences in task execution and remaining resources of different domains when the total number of tasks is 100. This paper chooses the total number of tasks of 100 as a reasonable task value suitable for the constructed scenario. The analysis finds that the Shannon entropy of the number of tasks to be executed in different domains is 2.92, demonstrating the uncertainty of the number of tasks. The satellite holding resources of some domains cannot fully satisfy the resource requirements for task execution, while domains 2 and 7 result in more resource surplus due to fewer tasks to be executed. As a result of inter-domain task collaboration, the unfinished tasks of domain 0 can be completed by the collaboration of domain 7. In addition, due to the presence of different task types, some domains may not be able to provide the execution of the subsequent tasks with minor resource requirements due to the over-consumption of a particular resource.

**Figure 5:**
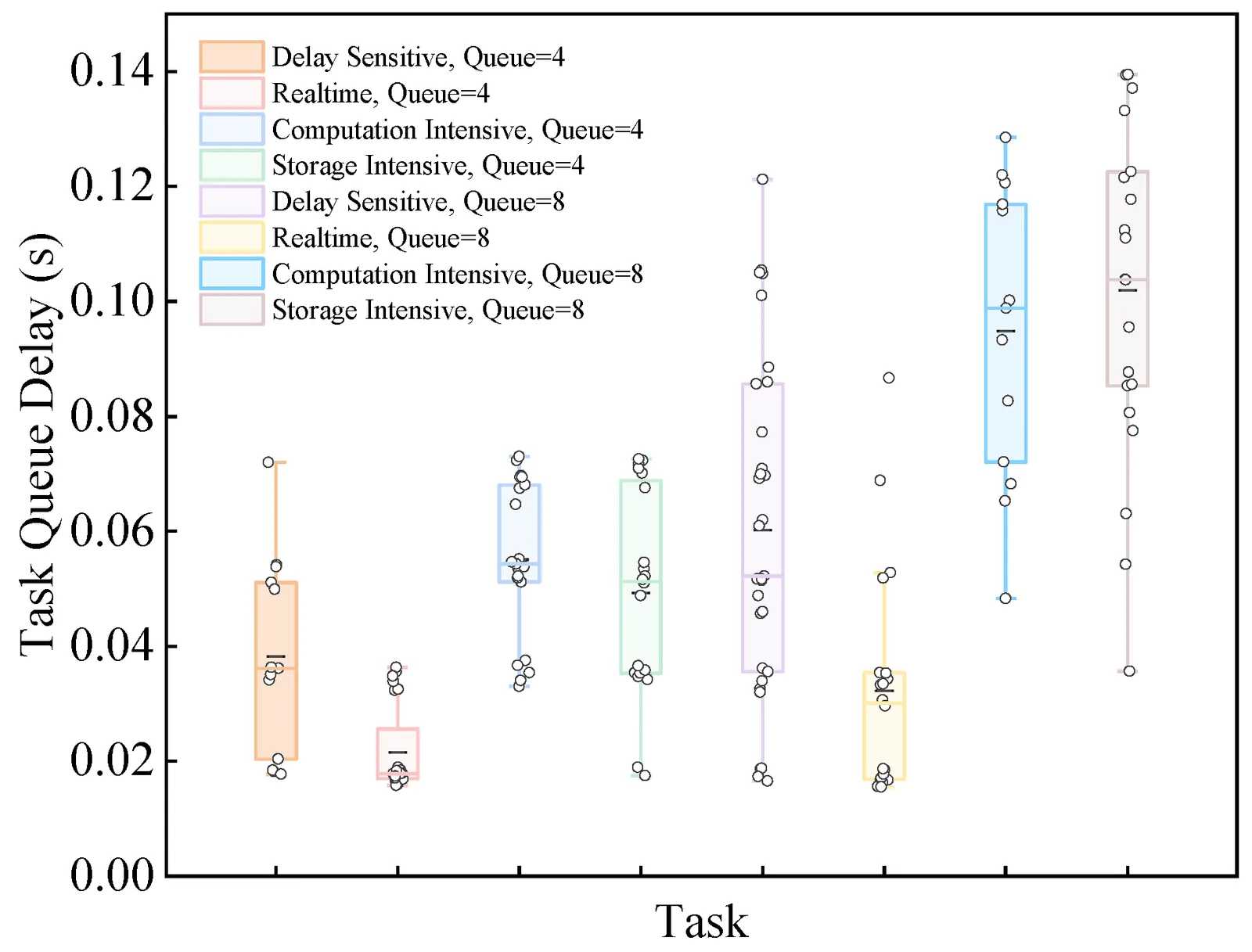
Queuing delay for each type of task at different task generation rates.

In order to study the execution efficiency of the proposed scheme for different types of tasks, we analyze the resource allocation priority of each type of task in terms of the variation of different task generation speeds. Figure 5 shows that under the same task generation speed, the execution efficiency of immediate and latency-sensitive tasks is better than that of computation-intensive and storage-intensive tasks, in which the execution efficiency of immediate tasks is the highest, with the smallest variance of statistical data. The maximal value of statistical data is similar to the statistical mean of latency-sensitive tasks, so half of the immediate tasks will get the highest resource allocation priority. The increase in task generation speed will correspondingly increase the queuing latency of various tasks. Accordingly, the queuing latency of instant and latency-sensitive tasks remains relatively small.

### 5.4. Comparative Performance Analysis

**Figure 6:**
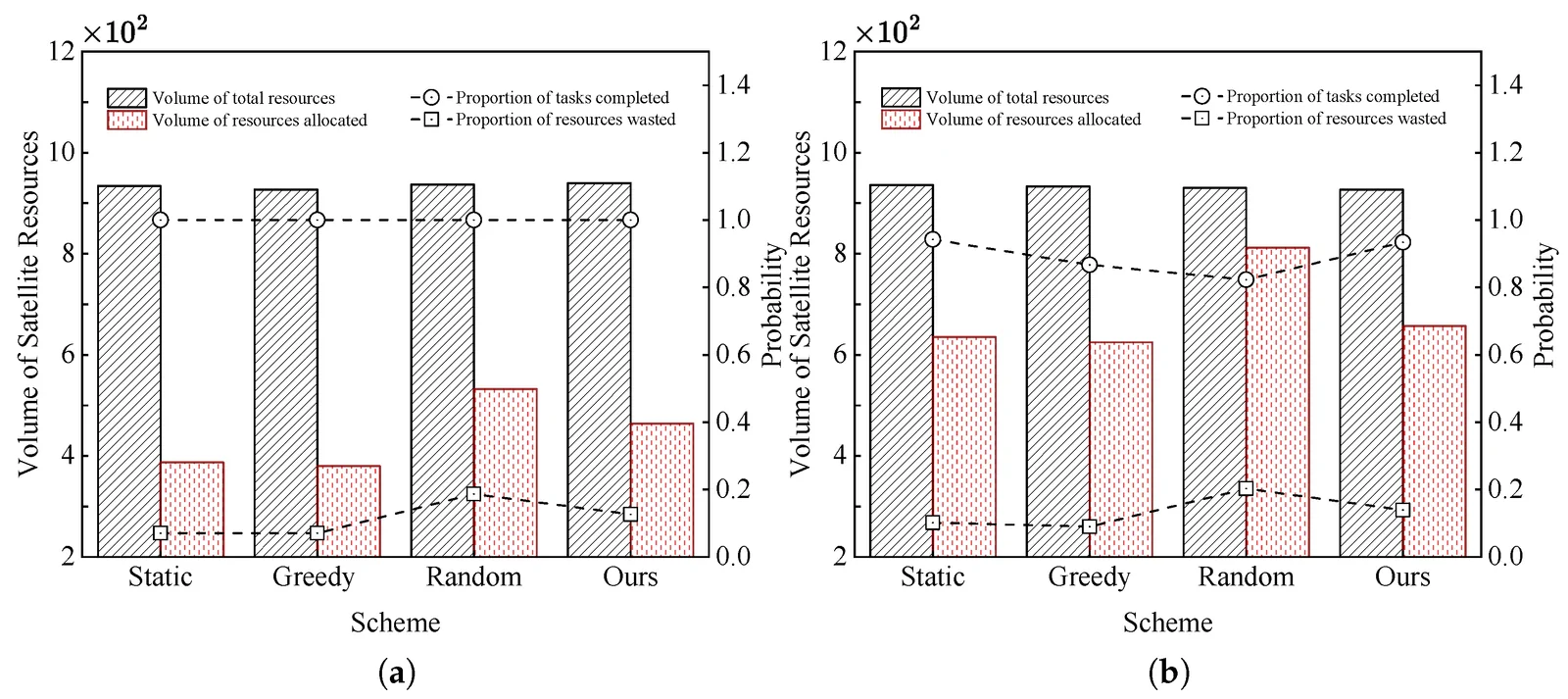
Comparison of task completion rates and resource utilization among schemes with different total number of tasks. (**a**) Number of tasks = 50. (**b**) Number of tasks = 100.

To further validate the satellite resource utilization of the proposed scheme, we study resource utilization when resources are fully allocated and under-allocated under different numbers of tasks, respectively. Narrowband interstellar communication is used in the construction scenario, and unrestricted interstellar collaboration will seriously increase the communication delay. In this paper, the resource allocation is based on the resource blocks held by individual satellites, and part of the resource utilization is sacrificed to alleviate the delay in system communication. When the total number of tasks is 50, different allocation strategies can satisfy the resource allocation requirements of tasks, in which the static strategy maintains the lowest resource wastage rate; here, because the static strategy can obtain the resource requirements of all the tasks to be allocated in advance, the resource wastage rate of the proposed scheme is slightly higher than that of the static strategy. When the number of tasks is 100, i.e., when the amount of resources held by the satellite is challenging to satisfy the task execution, the random strategy uses more satellite resources. However, it completes the task with the lowest rate. It is observed in Figure 6 that the resource wastage rate of the greedy strategy in this scenario is slightly reduced compared to the static strategy, which is analyzed to be the result of only greedily selecting the most suitable satellite resource blocks for the mission requirements while ignoring the long-term mission execution requirements. The proposed scheme maintains a better task completion rate while controlling the resource wastage rate in a similar range to the static strategy.

In the proposed framework, offline RL is employed for policy training, while the trained policy is deployed online for real-time resource management. We record some key performance metrics in Table 6 for the existing state-of-the-art cross-domain resource allocation schemes. Among them, the types and number of resources allocated by different schemes are inconsistent, so in the statistics, we only record the average utilization rate of each resource type, control the total number of domains to 10 for cross-domain task collaboration, and control the task density so that no task is left unexecuted during the allocation process. We set the total number of tasks for the proposed scheme to 50. All the comparison schemes use the reinforcement learning resource allocation scheme for online learning, except for the proposed scheme, which uses offline learning, and the inference latency of these comparison schemes is 3–5 times higher than the inference latency of the proposed scheme. However, the average resource utilization of the online learning version of the proposed scheme is higher, mainly due to the sustainable optimization of the online learning model, with a considerable degree of generalizability for immediate offline learning. In addition, the early-exit game matching method used by the proposed scheme achieves the early-exit of domains that satisfy the conditions for stable relationship establishment during the matching process, which can effectively reduce the time complexity of cross-domain collaborative matching and is highly valuable in similar narrow-band communication scenarios that require frequent data interactions.

We recorded the network performance metrics of the relevant schemes in Table 7. The same task generation model was used in the tests. The proposed scheme and MADDPG performed best and had comparable performance in terms of task completion rate and resource utilization. Compared with MADDPG, our scheme obtains a slightly higher task completion probability while maintaining a comparable resource utilization ratio.

## 6. Conclusions

In this paper, we study the frequent domain networking authentication pressure caused by high-speed satellite movement and the resulting dynamic resource management problem of unknown task flow under the uncertainty of domain-held resources, and we propose a dynamic cross-domain resource management scheme with joint network access authentication and task collaboration. For satellite networking authentication, the traditional encryption and decryption authentication process is improved to a non-interactive zero-knowledge evidence generation and verification process based on the satellite’s physical orbit information. For satellite access authentication, the traditional multi-round encryption-and-decryption process is replaced by a non-interactive zero-knowledge proof constructed from committed orbital attributes. Each accessing satellite aggregates its four orbital attributes into one proof, thereby reducing the overhead compared with four independent range proofs. Within the same domain and authentication epoch, the proof is transmitted once and can be independently verified by multiple authorized satellites. For concurrent access requests, different satellites generate independent proofs, while the verifier processes the received proofs through batch or parallel verification. Therefore, the proposed aggregation refers to multiple attributes of one satellite rather than joint proof generation by multiple satellites. In addition, for the dynamic resource allocation of unknown task flow, the multi-satellite cooperative resource allocation problem is modeled as a state-action-reward Markov decision process to maximize the local completion rate of tasks within the domain. An early-exit matching game strategy is designed to alleviate the resource imbalance between domains caused by task distribution’s spatial and temporal imbalance. The simulation results show that the inter-domain interaction communication overhead of the proposed scheme is only 38.6% of the symmetric encryption scheme, and a higher task completion rate is achieved with an average resource utilization rate of nearly 83%. In the future, we need to optimize the imbalance of mission distribution in partially observable satellite systems.

## Figures and Tables

**Table 1:** Computational overhead of actions involved in the authentication process.

Name of Operation Performed	Notation	Execution Time
One-way hash computation	$T_{h}$	0.034 ms
ECC-based scale multiplication	$T_{esm}$	0.427 ms
ECC-based point addition	$T_{epa}$	0.021 ms
Symmetry-based encryption	$T_{sec}$	0.029 ms
Symmetry-based decryption	$T_{sdc}$	0.053 ms

**Table 2:** Comparison of computational overhead for single authentication.

Scheme	Prover	Verifier	Total (ms)
Liu et al. [10]	$3T_{h}+5T_{esm}+2T_{epa}+T_{sec}$	$3T_{h}+5T_{esm}+2T_{epa}+T_{sdc}$	4.64
Ren et al. [35]	$9T_{h}+3T_{esm}$	$4T_{h}+3T_{esm}$	3.004
Guo et al. [36]	$5T_{h}+5T_{esm}+4T_{epa}$	$3T_{h}+4T_{esm}+2T_{epa}$	4.241
Zhang et al. [37]	$T_{h}+5T_{esm}+T_{epa}$	$T_{h}+3T_{esm}$	3.505
Our scheme	$3T_{h}+12T_{esm}+8T_{epa}$	$4T_{esm}+T_{epa}$	7.123

**Table 3:** Communication overhead for actions involved in the authentication process.

Name of Element Performed	Notation	Element Length
Identity	$L_{ID}$	64 bits
Timestamp	$L_{T}$	64 bits
One-way hash computation	$L_{H}$	128 bits
Symmetry-based encryption	$L_{SE}$	128 bits
ECC-based operation input/output	$L_{EO}$	320 bits
Element of the prime p	$L_{P}$	1024 bits
Orbital characterization information	$L_{O}$	8 bits
Message authentication code	$L_{AC}$	160 bits

**Table 4:** Theoretical comparison of single authentication communication overhead.

Scheme	Detailed Process	Total
Liu et al. [10]	$2L_{ID}+2L_{T}+5L_{SE}+2L_{P}$	2944
Ren et al. [35]	$L_{T}+L_{ID}+2L_{P}+2L_{SE}$	2432
Guo et al. [36]	$3L_{ID}+6L_{T}+3L_{EO}+7L_{AC}$	2656
Zhang et al. [37]	$L_{T}+L_{ID}+2L_{AC}+L_{P}+2L_{EO}$	2112
Our scheme	$L_{T}+L_{ID}+3L_{EO}+mL_{O}\cdot \log mL_{O}$	1136

**Table 5:** End-to-end authentication delay under different numbers of authenticated satellites.

Scheme	Number of Verifiers = 1	Number of Verifiers = 5	Number of Verifiers = 10	Number of Verifiers = 20	Number of Verifiers = 50
Liu et al. [10]	4.836	24.18	48.36	96.72	241.8
Ren et al. [35]	3.166	15.83	31.66	63.32	158.3
Guo et al. [36]	4.418	22.09	44.18	88.36	220.9
Zhang et al. [37]	3.646	18.23	36.46	72.92	182.3
Our scheme	7.199	7.586	8.093	9.074	12.16

**Table 6:** Comparison of the performance and overhead of related schemes.

Scheme	Resource Allocation Reasoning Latency (s)	Cross-Domain Task Collaboration Complexity	Average Resource Utilization Ratio
Mi et al. [30]	∖	$o\left(mn\right),\;m<n$	0.63
He et al. [38]	0.29	$o\left(m^{2}\right)$	0.76
Muhammad et al. [39]	0.32	∖	0.80
Ours (offline learning)	0.06	$o\left(mlogm\right)$	0.83
Ours (online learning)	0.23	$o\left(mlogm\right)$	0.91

**Table 7:** Comparison of network performance of related schemes.

Scheme	Greedy (Delay Priority)	Heuristic (Utilization Priority)	PPO	MADDPG	Ours
Task Completion Prob	0.537	0.765	0.804	0.914	0.921
Task Queueing Delay	0.09	0.23	0.18	0.21	0.11
Network Throughput	7.36	10.64	12.06	15.16	15.37
Resource Utilization Ratio	0.619	0.776	0.794	0.837	0.831

## Data Availability

The data that support the findings will be available in Bullerproof_ ZKP_Satellite at https://github.com/Qww0808/Bullerproof_ZKP_Satellite.git (accessed on 30 July 2026) following an embargo from the date of publication to allow for commercialization of research findings.
